# Mineral Content, Chemical Analysis, *In Vitro* Antidiabetic and Antioxidant Activities, and Antibacterial Power of Aqueous and Organic Extracts of Moroccan *Leopoldia comosa* (L.) Parl. Bulbs

**DOI:** 10.1155/2021/9932291

**Published:** 2021-07-20

**Authors:** Mohamed Boulfia, Fatima Lamchouri, Souad Senhaji, Nacima Lachkar, Khadija Bouabid, Hamid Toufik

**Affiliations:** Laboratory of Natural Substances, Pharmacology, Environment, Modeling, Health & Quality of Life (SNAMOPEQ), Polydisciplinary Faculty of Taza, Sidi Mohamed Ben Abdellah University of Fez, B.P. 1223 Taza-Gare, Taza, Morocco

## Abstract

Medicinal plants are a rich source of bioactive phytochemicals or bionutrients. Studies carried out during the past few decades have shown that these phytochemicals play an important role in preventing metabolic diseases such as cancer and diabetes. The present study was dedicated to the analysis of mineral and chemical composition and evaluation of antidiabetic, antioxidant, and antibacterial properties of aqueous and organic extracts of *Leopoldia comosa*, a plant with a long history of therapeutic and food use. Mineral content was determined using inductively coupled plasma atomic emission spectroscopy. Chemical composition was carried out by extraction of essential oils, preparation of aqueous and organic extracts, and qualitative and quantitative analysis. The biological study consisted of the evaluation of antidiabetic activity by inhibition of three enzymes, antioxidant activity by five tests, and antibacterial activity by the disc diffusion method. The correlation between chemical composition and antidiabetic and antioxidant properties was explored by PCA. The results showed that *L. comosa* contains high levels of Fe, K, P, Na, Cu, Mg, and Ca with values, respectively, in the order of 33552, 1843.14, 756.36, 439.65, 303.9, 272.37, and 20.55 mg/kg. Quantitative analysis showed that the diethyl ether extract had the highest content of polyphenols (129.75 ± 0.29 *µ*g GAE/mg E), flavonoids (988.26 ± 0.18 *µ*g QE/mg E), and tannins (30.22 ± 0.15 *µ*g CE/mg E). All extracts of *L. comosa* possess inhibitory activity of alpha-amylase, alpha-glucosidase, and beta-galactosidase enzymes, mainly the decocted and the acetone extract. The antioxidant results showed that organic extracts are more active than aqueous extracts especially diethyl ether extract which was similarly found to have an antibacterial effect on *Listeria innocua* and *Proteus mirabilis.* PCA allowed us to deduce that phenolic compounds, flavonoids, and tannins are strongly correlated with antioxidant and antidiabetic activity. *L. comosa* may have potential remedy in the prevention of metabolic disease.

## 1. Introduction

Biological systems are continuously exposed to oxidants, either generated endogenously by metabolic reactions or exogenously, such as air pollutants. Reactive oxygen species such as the superoxide anion (O_2_°) and the hydroxyl radical (OH), are very unstable species with unpaired electrons, capable of initiating the oxidation of proteins, lipids, and nucleic acids leading to alterations in cell structures and mutagenesis [1, 2]. Experimental studies have reported that the overproduction of free radicals with a deficiency of antioxidants is involved in the development of diabetes [3–5].

Parallel to oxidative stress, the evolution of our lifestyles, especially the modification of eating habits, with the overconsumption of fatty and sugary foods, coupled with a low intake of fruits and vegetables, plays a major role in the onset of diabetes. These dietary imbalances, combined with a lack of physical activity, lead to calorie intake over needs and energy storage in the adipose tissue. However, not all excess energy is stored in the form of fat, as some of the macronutrients are oxidized in the mitochondria, which promotes the production of free radicals [6].

Type 2 diabetes mellitus or non-insulin-dependent diabetes (NIDDM) is a multifactorial disease characterized by severe deregulation of glucose homeostasis. The World Health Organization (WHO) has predicted that between 2014 and 2045, the number of diabetics will double from 422 million to approximately 629 million people [7]. The incidence rate of NIDDM is higher in economically developed countries, particularly the US, where 9.1% of the population (29 million) has diabetes. In Morocco, between 2011 and 2015, the number of diabetics has increased from 1.5 million to more than 2 million, i.e., 25% more in 5 years. 80% of diabetes cases are type 2 [8].

In addition to the problem of oxidants, the rapid development of resistance in microbial agents and drug-induced side effects constitute a major public health problem, even in the most developed countries [9]. Indeed, the main determinant of the appearance of this resistance is probably the pressure of antibiotic selection to which microbial populations are subjected. Thus, for all these reasons, research is now focusing on new therapeutic alternatives such as medicinal plants that have been used for centuries in the treatment of many diseases. Active components responsible for antioxidant, hypoglycemic, and antibacterial activity may include polysaccharides, ascorbic acid (vitamin C), carotenoids, triterpenoids, alkaloids, flavonoids, coumarins, phenolic substances, and peptides [6].

Within the framework of the investigations of the phytochemical properties and valorization of the pharmacological activities of the natural substances of the Taza region, carried out by our laboratory: natural Substances, Pharmacology, Environment, Modelling, Health and Quality of Life (SNAMOPEQ) [10–15], we have selected for the present study a medicinal plant named *Leopoldia comosa* (L.) commonly called “Bssilla” which despite its use in traditional Moroccan medicine for its therapeutic properties [16] and its exploitation for its economic value has not been investigated either to study its chemical composition or to evaluate its pharmacological properties either in Morocco or in Southern Mediterranean countries. Indeed, in our previous preliminary work related to the ethnomedicinal and socioeconomic value that we have conducted from March 2018 to May 2019 in the province of Taza, Morocco has shown that this plant is used in the treatment of dermatological and digestive disorders. It also represents an important source of income for the population and farmers of the plant in the region of Taza, especially as Morocco is involved in the export of this plant abroad, particularly to Italy as this plant is used by Italians in food and the treatment of many diseases [16].

Hence, the interest of this study was to evaluate for the first time the mineral composition of the bulb of Moroccan *L. comosa* and to conduct phytochemical studies by a qualitative and quantitative analysis of secondary metabolites (alkaloids, polyphenols, flavonoids, tannins, anthraquinones, anthracenosides, quinones, saponins, and sterols) and pharmacological studies by the evaluation of antidiabetic, antioxidant, and antibacterial biological activities. The antidiabetic activity was studied by three assays using the enzymes responsible for inhibiting polysaccharide degradation; alpha-amylase, alpha-glucosidase, and beta-galactosidase; antioxidant activity by five different methods (H_2_O_2_, ABTS, DPPH, FRAP, and RP); and antibacterial activity by the disc diffusion method. A principal component analysis (PCA) was also performed to investigate the correlation between the contents of phenolic compounds and the results of the *in vitro* tests for antidiabetic and antioxidant activities.

## 2. Materials and Methods

### 2.1. Plant Material

*Leopoldia comosa* (L.) bulbs were harvested in the province of Taza, Morocco (geographic coordinates: N 34°13.605′ W 004°01.711′, altitude: 469 m) during the spring, March 2018, and the plant was identified by Dr. Abdelmajid Khabbach, the botanist of the Laboratory of Natural Substances, Pharmacology, Environment, Modelling, Health and Quality of Life (SNAMOPEQ), Polydisciplinary Faculty of Taza (FPT), Sidi Mohamed Ben Abdellah University of Fez, Morocco. A voucher specimen was deposited in the herbarium under the code SA 2018/05. The bulb was cleaned, peeled, and left to dry in the shade and at room temperature. The full name of the plant was taken as described on the website (https://www.theplantlist.org; *Leopoldia comosa* (L.) Parl.).

### 2.2. Mineral Content of *Leopoldia comosa* (L.)

The analysis of the mineral composition of *L. comosa* bulbs (potassium (K), calcium (Ca), magnesium (Mg), sodium (Na), phosphorus (P), copper (Cu), iron (Fe), selenium (Se), strontium (Sr), and zinc (Zn) was determined using inductively coupled plasma atomic emission spectroscopy ((ICP-AES) HORIBA JOBIN YVON) as previously described [17]. Thus, 0.5 mg of *L. comosa* bulbs was digested with nitric acid and perchloric acid (25%: 75%) solution, before being incinerated at 110°C, and then brought back dry until the mineralization was discolored on a sand bath. The residue was dissolved in 10 mL HCL (5%), and the contents were filtered through 0.45 *µ*m porosity filters until a clear solution was obtained. The sample solution was made up to a final volume of 25 mL with distilled water and analyzed by atomic absorption spectrophotometry.

### 2.3. Preparation of Extracts

#### 2.3.1. Aqueous Extraction

It is a method of preparation using distilled water in three modalities that vary according to temperature and extraction time. The aqueous extracts were prepared according to the methodology described previously in our work [10, 13–15]:

1. *Decoction.* 10 g of the plant material was mixed with 100 mL of distilled water in a ground glass flask, topped with a condenser, and left to boil at a stable boiling temperature for 20 minutes.
2. *Infusion.* 100 mL of boiling distilled water was poured onto 10 g of the plant material in a beaker for 30 minutes.
3. *Maceration.* 10 g of the plant material was mixed with 100 mL of cold distilled water in a beaker for 24 hours.

After filtration, the 3 prepared aqueous extracts were frozen at (−80°C) for 24 hours, lyophilized using a (Heto PowerDry LL3000).

#### 2.3.2. Organic Extraction

The organic extracts were prepared by two different methods; the first technique is realized under hot conditions using a Soxhlet apparatus, where 100 g of plant material was introduced into a cartridge of cellulose attached to a ball and surmounted by a refrigerant and 1000 mL of three solvents of increasing polarity (diethyl ether, acetone, and ethanol) used separately was vaporized and then condensed while remaining in contact with the plant material. The extraction is ended when the solvent of extraction becomes clearer, six hours for our experimental conditions. The second technique was cold maceration by pouring 1000 ml of the solvents described previously separately on 100 g of the plant material for 48 hours.

Organic extracts were evaporated on a rotary evaporator (Büchi AG CH-9230) under vacuum at 40–50°C and stored with the aqueous extracts at 4°C for uses in phytochemical and pharmacological studies.

#### 2.3.3. Extraction of Essential Oils (EOs) by Hydrodistillation

The essential oils (EOs) were extracted by hydrodistillation using a Clevenger apparatus. The extraction was done twice, with fresh bulbs of *L. comosa* and the second time after drying the bulbs at room temperature. The extraction consisted of immersing 100 g of the bulbs (fresh or dried) in a flask filled with one liter of distilled water, which was then brought to a boil.

### 2.4. Phytochemical Analysis

#### 2.4.1. Qualitative Analysis of Phytochemicals

Phytochemical screening tests allow us to characterize the presence or absence of secondary metabolites through a qualitative analysis based on coloration and/or precipitation reactions. In this study, the search for different secondary metabolites, such as alkaloids, tannins, saponins, anthracenosides, anthraquinones, quinones, flavonoids, and sterols, was carried out on the plant bulb and the nine aqueous and organic extracts prepared from *L. comosa* as described in the previous work of our laboratory [10, 13–15].

#### 2.4.2. Quantitative Analysis of Phytochemicals

The dosage of secondary metabolites in the *L. comosa* bulbs was conducted according to the results of the phytochemical screening tests. Thus, the content of polyphenols, flavonoids, and tannins was determined. The assay was carried out as described in previous work in our laboratory [12–15].

*(1) Determination of Polyphenol Content*. The Folin–Ciocalteu method [18] was used to determine the total phenolic content of our extracts. A volume of 0.5 mL of each of our nine aqueous and organic extracts or gallic acid was introduced into test tubes, 2.5 mL of Folin–Ciocalteu reagent was added, and then 4 mL of 7.5% (m/v) sodium carbonate was added. The different solutions were kept in a water bath for 30 minutes. Absorbance was measured at 765 nm using a SPECUVIS2 UV/Vis Spectrophotometer, No: HF1309003. The polyphenol content in the extracts was expressed in microgram (*μ*g) gallic acid equivalent per milligram (mg) of extract (*μ*g GAE/mg E).

*(2) Determination of Flavonoid Content*. The quantification of flavonoids in aqueous and organic extracts of *L. comosa* was carried out by the colorimetric method of aluminum chloride AlCl_3_, based on the protocol described by Dewanto et al. [19] and as presented in our previous work [13]. The flavonoid content was expressed as *μ*g quercetin equivalent/mg of extract (*µ*g QE/mg E).

*(3) Determination of Tannin Content*. The content of tannins in *L. comosa* extracts was determined by the vanillin method according to the protocol of [20] and as presented in our previous work [13]. A volume of 50 *μ*L of each sample of our nine aqueous and organic extracts or catechin was added to 1500 *μ*L of the vanillin/methanol solution (4%, w/v) and then mixed using a vortex. Then, 750 *μ*L of concentrated hydrochloric acid (HCl) was added and allowed to react at room temperature for 20 minutes. The absorbance was measured at 500 nm, and the concentration of tannins was expressed in microgram (*μ*g) catechin equivalents per milligram (mg) of extract (*μ*g CE/mg E).

### 2.5. Biological and Pharmacological Assays

#### 2.5.1. Study of Antidiabetic Activity

*(1) Alpha-Amylase Inhibitory Assay*. The alpha-amylase inhibition assay was performed using the 3, 5-dinitrosalicylic acid (DNSA) method [21]. Different concentrations of extracts from the bulb of *L. comosa* were prepared in saline phosphate buffer (Na_2_HPO_4_/NaH_2_PO_4_ (0.02 M) at pH 6.9). A volume of 200 *μ*L of alpha-amylase solution (2 units/mL) was mixed with 200 *μ*L of the extract and was incubated for 10 min at 30°C. Thereafter, 200 *μ*L of the starch solution (1% in water (w/v)) was added to each tube and incubated for 3 min at 30°C. The reaction was terminated by the addition of 200 *μ*L DNSA reagent (12 g of sodium potassium tartrate tetrahydrate in 8.0 mL of 2 M NaOH and 20 mL of 96 mM of 3, 5-dinitrosalicylic acid solution) and was boiled for 10 min in a water bath at 85–90°C. The mixture was cooled to ambient temperature and was diluted with 5 mL of distilled water, and the absorbance was measured at 540 nm using a SPECUVIS2 UV/Vis spectrophotometer, no: HF1309003. Acarbose was used as a positive control.

The *α*-amylase inhibitory activity was expressed as percent inhibition and was calculated using the following equation:

(1) $$\begin{array}{c} \text{inhibition}\left(\%\right)=100\times \left[\frac{\left(\text{Ac}-\text{Acb}\right)-\left(\text{As}-\text{Asb}\right)}{\left(\text{Ac}-\text{Acb}\right)}\right], \end{array}$$

where Ac refers to the absorbance of the control (enzyme and buffer), Acb refers to the absorbance of control blank (buffer without enzyme), As refers to the absorbance of the sample (enzyme and extract), and Asb is the absorbance of sample blank (extract without enzyme). The concentration of extract providing 50% inhibition (IC50) was calculated from the calibration curve.

*(2) Alpha-Glucosidase Inhibitory Assay*. The inhibitory potency of aqueous and organic extracts of *L. comosa* against alpha-glucosidase enzyme was evaluated by measuring the formation of 4-nitrophenol by alpha-glucosidase after reaction with 4-p-nitrophényl-*α*-D-glucopyranoside (pNPG) according to the method of Lordan et al. [22]. To perform this test, a reaction mixture containing 150 *µ*L of extracts prepared in sodium phosphate buffer (0.1 M/pH = 6.7) at various concentrations and 100 *µ*L of *α*-glucosidase solution (0.1 U/mL) was preincubated at 37°C for 10 min. Subsequently, 200 *µ*L of 1 mM of p-nitrophényl-*α*-D-glucopyranoside (pNPG) solution in sodium phosphate buffer (0.1 M/pH = 6.7) was added and incubated at 37°C for 30 min. The reaction was terminated by adding 1 mL of sodium carbonate solution (Na_2_CO_3_/0.1 M) and the absorbance was measured at 405 nm. Acarbose was included as a positive control, and the percentage inhibition was determined as described in the alpha-amylase assay, and the IC50 values were determined.

*(3) Beta-Galactosidase Inhibitory Assay*. The *in vitro* assessment of antidiabetic activity by inhibition of beta-galactosidase is a test based on the arrest of beta galactoside degradation by inhibition of intestinal *β*-galactosidase activity. Indeed, the beta-galactosidase or lactase is an enzyme capable of hydrolyzing lactose by transforming it into glucose and galactose [10]. For this purpose, a mixture of 150 *μ*L of different concentrations of the extracts and 100 *μ*L of sodium phosphate buffer (0.1 M at pH = 7.6) containing the enzyme solution beta-galactosidase (1 U/mL) was incubated at 37°C for 10 min. Then, 200 *μ*L of the substrate 2-nitrophenyl *β*-D-galactopyranoside (1 mM) solubilized in sodium phosphate buffer was added. The reaction mixtures were incubated at 37°C for 30 min. After incubation, 1 mL Na_2_CO_3_ was added to stop the reaction and the absorbance was recorded at 410 nm using a spectrophotometer. Quercetin was used as a positive control and the percentage inhibition was determined as described in the alpha-amylase assay, and the IC50 values were determined.

#### 2.5.2. Study of Antioxidant Activity

Various methods were adopted to assess the antioxidant activity *in vitro* of *L. comosa* extracts, namely, hydrogen peroxide scavenging assay (H_2_O_2_), ABTS or TEAC (equivalent antioxidant capacity of Trolox), DPPH (2, 2-diphenyl-1-picrylhydrazyl), ferric reducing antioxidant power assay (FRAP), and reducing power (RP).

*(1) Hydrogen Peroxide Scavenging Assay (H*_*2*_ *O*_*2*_). The ability of *L. comosa* aqueous and organic extracts to scavenge H_2_O_2_ was determined using the method of Ruch et al. [23]. A hydrogen peroxide solution (40 mM) was prepared in a solution of phosphate saline buffer (PBS, pH 7.4). The concentration of hydrogen peroxide was determined after 10 minutes by absorption at 230 nm using a spectrophotometer.

The percentage scavenging of H_2_O_2_ by our extracts and ascorbic acid was determined according to the equation:

(2) $$\begin{array}{c} \left(\%\right)=\left[\frac{\left(\left(\text{Ac}\right)|\left(\text{As}-\text{Asb}\right)\right)}{\text{Ac}}\right]\times 100, \end{array}$$

where Ac is the absorbance of the control (H_2_O_2_ + phosphate-buffered saline), As is the absorbance of the sample (H_2_O_2_ in phosphate-buffered saline + extract), and Asb is the absorbance of the blank (extract + phosphate-buffered saline) [13].

*(2) Trolox Equivalent Antioxidant Capacity Using ABTS (TEAC)*. The antioxidant activity of the nine prepared extracts was determined according to the protocol of Re et al. [24] and as presented in our previous work [13]. The stock solution was prepared by mixing an ABTS solution of (7 mM) with potassium persulfate (2.45 mM), and the mixture was left in the dark at room temperature for 12–16 hours before use. 30 *μ*L of our extracts were reacted with 3 mL of the ABTS^+^ solution, and the absorbance was measured at 734 nm after 1 min using a spectrophotometer. Trolox was used as the reference standard, and results were expressed in *µ*g Trolox equivalent per milligram of extract (*µ*g TE/mg E).

*(3) DPPH (2, 2-Diphenyl-1-picrylhydrazil) Free Radical Scavenging Activity*. The chemical compound 2, 2-diphenyl-1-picrylhydrazyl (DPPH) was one of the first free radicals used to study the relationship between the structure and antiradical activity of phenolic compounds. It has an unpaired electron on an atom of the nitrogen bridge [25]. To make this test, 3 mL of different concentrations of our extracts were added to 1 mL of the DPPH solution (200 *µ*M) and the mixture was left in the dark for 30 min at 30°C, and the absorbance was measured at 517 nm using a spectrophotometer. Trolox, BHT, and ascorbic acid were used as the reference standard.

*(4) Ferric Reducing-Antioxidant Power Assay (FRAP)*. The antioxidant power of iron reduction (FRAP) was used to measure the ability of extracts to reduce the TPTZ-Fe (III) complex to TPTZ-Fe (II) measured at wavelength 593 nm [26]. 100 *µ*L of the *L. comosa* extracts was reacted with 3000 *µ*L of the FRAP solution (25 mL acetate buffer, 2.5 mL TPTZ, and 2.5 mL FeCl_3_·6H_2_0) for 30 minutes in the dark. The results were expressed in *µ*g Trolox equivalent per milligram of extract (*µ*g TE/mg E) [13].

*(5) Reducing Power Assay (RP)*. The RP assay was developed to measure the ability of the extracts tested to reduce ferric iron (Fe^3+^) present in the potassium ferricyanide complex K_3_Fe (CN) 6 to ferrous iron (Fe^2+^). Based on the protocol developed by Oyaizu in 1986 [27] and as described in our publication [13], results were expressed in *µ*g ascorbic acid equivalent per milligram of extract (*µ*g AAE/mg E).

#### 2.5.3. Study of Antibacterial Activity

*(1) Bacterial Strains*. The antibacterial activity of organic extracts of *L. comosa* was tested against six reference bacterial strains; these are pathogenic bacteria frequently involved in infectious diseases. Three bacteria are Gram-positive, *Staphylococcus aureus* (CECT976), *Bacillus subtilis* (DSM6633), and *Listeria innocua* (CECT 4030), and three Gram-negative bacteria, *Escherichia coli* (K12), *Proteus mirabilis*, and *Pseudomonas aeruginosa* (CECT118).

*(2) Inoculum Preparation*. The inoculum suspension was obtained by taking colonies from 24 hours' cultures. The colonies were suspended in a sterile aqueous solution of NaCl (0.9%) and shacked for 20 seconds. The density was adjusted to the turbidity of a 0.5 McFarland Standard (10^8^ CFU/mL, colonies forming a unit per mL).

*(3) Agar Disc Diffusion Assay*. Antibacterial activity of organic extracts of *L. comosa* was determined by the agar disc diffusion assay according to the method described by Sharififar et al. [28]. A suspension of microorganisms from an inoculum of 10^8^ CFU/mL was inoculated on the surface of agar plates containing 20 mL of Mueller Hinton Agar using a sterile swab. Sterile discs (6 mm in diameter) soaked in different concentrations of the extracts prepared from *L. comosa* bulbs (40, 80, and 100 mg/mL) solubilized in DMSO (10%) were placed on the surface of the agar plate. Then, the plates were closed and incubated at 37°C for 20 hours. The antibacterial effect of our extracts was evaluated by measuring the zone of inhibition formed around the discs and expressed in mm against the six bacterial strains tested. Negative control was produced by DMSO (10%) while the positive control is represented by the two antibiotics tetracycline and amikacin. All tests were repeated three times, and the results were calculated as follows: mean ± standard deviation.

### 2.6. Statistical Analysis

The results were expressed as the mean ± standard error. Nonlinear regression analysis was adopted to determine the IC50 values of the four assays (alpha-amylase, alpha-glucosidase, beta-galactosidase, and DPPH assays). The data were analyzed by one-way analysis of variance (one-way ANOVA), Turkey: compare all pairs of column procedure for the significance of the difference. A difference in the mean values of *P* < 0.05 was considered to be statistically significant. The analysis was performed with GraphPad Prism® 5.0 software. Principal component analysis (PCA) was performed by the XLSTAT software.

## 3. Results

### 3.1. Mineral Composition of *Leopoldia comosa* (L.) Bulbs

According to our bibliographic research, we did not find any studies relating to the evaluation of the mineral composition and nutritional value of *L. comosa.* The results of the analysis of the mineral composition of *L. comosa* in our study represent the first investigation conducted in this plant. This shows that the *L. comosa* bulb has high levels of Fe (33552), K (1843.14), P (756.36), Na (439.65), Cu (303.9), Mg (272.37), and Ca (20.55) mg/kg of plant material. However, lower values were noted for the 3 elements Se, Sr, and Zn (<0.01 mg/L).

### 3.2. Phytochemical Study of *Leopoldia comosa* (L.) Bulbs

#### 3.2.1. Yields of Aqueous and Organic Extractions of *Leopoldia comosa* (L.) Bulbs

The extraction yield is the ratio between the weight of the compounds or substances that can be extracted depending on the nature of the solvent used, the extraction method, and the nature of the plant material used, whether dry or fresh. The yield is expressed as a percentage and is calculated by the following formula:

(3) $$\begin{array}{c} R=\frac{\text{PA}}{\text{PB}}\times 100, \end{array}$$

where A is the extraction yield in (%), PA is the weight of compound in g, and PB is the weight of dry plant material in g.

The yields obtained were highly variable, ranging from 0.2 to 8%. The highest yield was obtained for the most polar solvent, water, with the decoction modality with a percentage of 8%, followed by the infused extract and the aqueous macerate with values of about 7% and 5.2%, respectively, whereas organic solvents have low values, whose highest yield was obtained with the most polar solvents, namely, ethanol, acetone, and diethyl ether, and with cold extraction by maceration with values of about 1.3%, 0.8 %, and 0.5%, respectively. Ethanolic, acetone, and diethyl ether extracts prepared by Soxhlet showed values of about 1%, 0.6%, and 0.2%, respectively.

#### 3.2.2. Extraction Yields of Essential Oils (EOs) from *Leopoldia comosa* (L.) Bulbs

The results of the investigations of the extraction of essential oils from *L. comosa* bulb whether fresh or dried allowed us to note an absence of essential oils in this part of the plant.

#### 3.2.3. Phytochemical Screening

Phytochemical screening was carried out on *L. comosa* in two parts: first on the plant bulbs and the second on the aqueous and organic extracts prepared. The results obtained revealed the presence of flavonoids, catechin tannins, and quinones in the case of the plant bulbs and the 9 aqueous and organic extracts prepared. The families of anthracenosides and anthraquinones are present in the bulb and organic extracts while they are absent in the aqueous extracts.

#### 3.2.4. Polyphenol, Flavonoid, and Tannin Contents

Phenolic compounds are the most diverse compounds of secondary metabolites found in plant organs, which can be used as therapeutic agents, preservatives, additives, and food supplements [29].  Table 1 summarizes the results obtained for the polyphenol, flavonoid, and tannin contents of the aqueous and organic extracts of the *L. comosa* bulbs.

According to Table 1, we noticed that the contents of phenolic compounds, flavonoids, and tannins vary according to the aqueous or organic extraction method and the hot or cold extraction modality.

Aqueous extracts showed lower contents of phenolic compounds than the organic extracts with a significant difference (*P* < 0.05). For aqueous extracts, the difference was nonsignificant (*P* < 0.05) between decocted, infused, and macerated and showed values of 4.28 ± 0.02, 4.44 ± 0.02, and 4.69 ± 0.01 *µ*g GAE/mg E, respectively. For organic extracts, the difference is significant between the six prepared extracts (*P* < 0.05). The diethyl ether extract prepared by Soxhlet with the least polar solvent showed the highest value which is of the order of 129.75 ± 0.29 *µ*g GAE/mg E, followed by the macerated diethyl ether extract, acetone extract, macerated acetone extract, ethanolic extract, and lastly the ethanolic extract prepared by maceration with values of the order of 115.81 ± 0.24, 69.96 ± 0.01, 61.43 ± 0.04, 20.49 ± 0.08, and 18.20 ± 0.04 *µ*g GAE/mg E, respectively.

For the flavonoid content, the aqueous extract prepared by the infusion mode was found to be richer than the decocted and macerated with values on the order of 90.82 ± 0.59, 82.15 ± 0.26, and 78.63 ± 0.21 *µ*g QE/mg E, respectively, with a significant difference (*P* < 0.05) between decocted and infused, and infused and macerated, and the no significant difference between decocted and macerated. For organic extracts, always hot extraction makes it possible to extract more flavonoids than cold extraction used by the same solvent, in particular for diethyl ether extract followed by acetone and ethanolic extracts with values of the order of 988.26 ± 0.18, 330.15 ± 1.45, and 147.63 ± 0.57 *µ*g QE/mg E, respectively.

In the case of tannins, we obtained the highest content with the diethyl ether extract prepared by Soxhlet (30.22 ± 0.15 *µ*g CE/mg E), and in the case of aqueous extracts, the macerate gives the best yield (18.68 ± 0.11 *µ*g CE/mg E).

### 3.3. Biological and Pharmacological Assays

#### 3.3.1. Antidiabetic Activity

*(1) Alpha-Amylase Inhibitory Assay*. The results of the evaluation of the alpha-amylase inhibitory activity of aqueous and organic extracts from the bulb of *L. comosa* are shown in Table 2. IC50 values were calculated for all aqueous and organic extracts and the reference standard, and a lower IC50 value indicates a higher inhibitory activity. The results obtained showed that the aqueous extracts and particularly the decocted extract have a high alpha-amylase inhibitory capacity with an IC50 of 1200.66 ± 13.79 *µ*g/mL, which is twice better than the macerated extract with an IC50 of 2752.33 ± 8.11 *µ*g/mL. Similarly, for organic extracts, we have recorded that hot extraction by Soxhlet gives interesting results compared to cold extraction by maceration, of which ethanolic extract, acetone extract, and diethyl ether extract had IC50s, respectively, of 2264 ± 22.86 *µ*g/mL, 2219.33 ± 3.31 *µ*g/mL, and 2512.33 ± 5.98 *µ*g/mL versus IC50 values of 2384 ± 7.40 *µ*g/mL, 2289.66 ± 7.45 *µ*g/mL, and 2897.66 ± 4.76 *µ*g/mL, respectively, for the same extracts prepared by cold maceration. A highly significant difference was observed for all extracts and the reference standard, acarbose, which had an IC50 of 616.33 ± 6.58 *µ*g/mL.

*(2) Alpha-Glucosidase Inhibitory Assay*. To explore the antidiabetic activity of aqueous and organic extracts of *L. comosa*, the alpha-glucosidase inhibition assay was performed and the results are shown in Table 2. According to this table, all the extracts tested showed an interesting hypoglycemic property with IC50s ranging from 85.41 ± 3.86 *µ*g/mL to 268.23 ± 2.85 *µ*g/mL. The acetone extract was most active with an IC50 of 85.41 ± 3.86 *µ*g/mL which is significantly lower than the reference standard, acarbose (IC50 = 195 ± 5 *µ*g/mL). For the aqueous extracts, the decocted was the active extract with an IC50 of 238.53 ± 2.35 *µ*g/mL against an IC50 of 268.23 ± 2.85 *µ*g/mL for the cold prepared macerated extract. In this test, hot extraction seems to be the best method to obtain the great hypoglycemic power of aqueous and organic extracts of *L. comosa* bulb.

*(3) Beta-Galactosidase Inhibitory Assay*. Based on our literature search, this study presents for the first time the results of beta-galactosidase inhibition. According to Table 2, we note that organic extracts are more active than aqueous extracts, with a better activity obtained by the acetone extract with an IC50 of the order of 163.5 ± 2.51 *µ*g/mL. For the aqueous extracts, the decocted recorded an IC50 value of 205.43 ± 2.22 *µ*g/mL against 245.5 ± 9.26 *µ*g/mL for the macerated extract. These results are in line with those of the alpha-amylase and alpha-glucosidase inhibition assay, for which we found that hot extraction is the best method for preparing the extracts responsible for inhibiting the enzymes of the antidiabetic activity.

Several previous studies have reported that diabetes is associated with oxidative stress [3–5], and this through the accumulation of free radicals that can lead to changes in the genetic material of the cell and thus modify its metabolic functioning. Similarly, the chronic hyperglycemic state of diabetes mellitus leads to oxidative stress including several mechanisms such as the auto-oxidation of glucose leading to the formation of the superoxide anion radical and activation of the hexosamine pathway. For these reasons, we continued our study and tested the antioxidant activity of aqueous and organic extracts of *L. comosa* to determine their ability to scavenge free radicals using five different and complementary tests with different mechanisms (H_2_O_2_, ABTS, DPPH, FRAP, and RP).

#### 3.3.2. Antioxidant Activity

In this study, the antioxidant potential of the 9 aqueous and organic extracts from the bulb of *L. comosa* was determined by five methods, and the results of the H_2_O_2_, ABTS, DPPH, FRAP, and RP tests are presented in Table 3 and show that all the extracts prepared from *L. comosa* bulbs possess significant antioxidant properties.

*(1) Hydrogen Peroxide Scavenging Assay (H_2_O_2_)*. Table 3 presents for the first time the results of hydrogen peroxide scavenging of *L. comosa* bulb extracts and those of ascorbic acid used as the reference standard. Aqueous extracts showed great activity by this test with a high percentage of scavenging of H_2_O_2_, respectively, of the order of 62.12 ± 0.2%, 61.89 ± 0.3%, and 61.72 ± 0.1% for decocted, infused, and macerated with a no significant difference (*P* < 0.05) between these three aqueous extracts. Organic extracts also showed better H_2_O_2_ scavenging activity, and the hot diethyl ether extract prepared by Soxhlet was the most active with a percentage of 62.67 ± 0.06%, followed by the two hot prepared ethanolic and acetone extracts with a percentage of 61.3 ± 0.16% and 61.24 ± 0.2%. The hot-prepared diethyl ether extract was the only one that showed a no significant difference (*P* < 0.05) with the reference standard (ascorbic acid) which showed a percentage of 63.63 ± 0.47%.

*(2) Trolox Equivalent Antioxidant Capacity Using ABTS (TEAC)*. Trolox equivalent antioxidant capacity is based on the inhibition of ABTS ^+^ radical solution absorbance when it is exposed to an antioxidant. It should be noted that the higher the TEAC value, the more active the molecule is. The results of the ABTS test are expressed in *µ*g Trolox equivalent per milligram of extract (*μ*g TE/mg E) (Table 3). Antioxidant capacity is classified in the following order: diethyl ether > acetone > macerated diethyl ether > macerated acetone > ethanolic > macerated ethanolic > decocted > infused > and aqueous macerate last.

*(3) 2, 2-Diphenyl-1-picrylhydrazil Free Radical Scavenging Activity (DPPH)*. To study the antiradical activity of our 9 aqueous and organic extracts, we evaluated their ability to scavenge the free radical DPPH°. DPPH° is a free radical that accepts an electron or hydrogen radical to become a stable molecule, and its purple color shows a characteristic absorption at 517 nm. The results of this test are expressed in IC50 illustrated in Table 3. The scavenging effect of the DPPH radical showed an activity that is dependent on the nature of the solvent employed, the extraction modality, and the concentration tested.

The results of this test showed that for the aqueous extracts, the decocted and the infused have IC50 values of 1011.33 ± 4.37 and 1089.33 ± 0.92 *µ*g/mL, respectively, with a no significant difference and that they remain more active than that of the aqueous macerate (1140 ± 20.64 *µ*g/mL). The organic extracts showed higher activity compared to the aqueous extracts with an IC50 of 10.08 *µ*g/mL for diethyl ether extract, followed by macerated diethyl ether extract, acetone extract, macerated acetone, ethanolic extract, and macerated ethanol with IC50s, respectively, of the order of 10.15 ± 0.04, 99.76 ± 0.04, 100 ± 0.03, 139.4 ± 6.93, and 220.5 ± 2.91 *µ*g/mL, with a significant difference between ethanolic extract, macerated ethanolic extract, acetone extract, and a no significant difference between the diethyl ether extract prepared by hot and cold modalities; likewise, for hot and cold acetone extract, the difference is no significant.

*(4) Ferric Reducing-Antioxidant Power Assay (FRAP)*. The antioxidant power of *L. comosa* extracts was estimated from their ability to reduce the TPTZ-Fe (III) complex to TPTZ-Fe (II) measured at wavelength 593 nm. The results are expressed in *µ*g Trolox equivalent per milligram of extract (*μ*g TE/mg E).

The results obtained are presented in Table 3, and we noticed that all the tested extracts have a strong capacity for reducing iron with a significant difference (*P* < 0.05) between the aqueous and organic extracts and that the organic extracts are more active than aqueous extracts whose difference between decocted and infused is not significant, represented by values of 12.9 ± 0.1 and 11.16 ± 0.52 *µ*g TE/mg E, respectively. For organic extracts, the diethyl ether extract is the most active with a value of 394.77 ± 0.74 *µ*g TE/mg E, followed by the macerated diethyl ether, acetone, macerated acetone, ethanolic, and macerated ethanolic extract with values, respectively, of the order of 358.77 ± 0.74, 277.74 ± 067, 225.77 ± 0.15, 131.55 ± 0.26, and 49.24 ± 0.13 *µ*g TE/mg E. The organic extracts showed a highly significant difference (*P* < 0.05) between them.

*(5) Reducing Power Assay (RP)*. In this test, the yellow color of the test solution changes to different shades of green and blue, depending on the reducing power of each compound. A higher absorbance at 700 nm indicates a higher reducing power of the extract. The results are expressed in *µ*g ascorbic acid equivalent per milligram of extract (*µ*g AAE/mg E). The reducing power of our 9 prepared extracts is shown in Table 3. A highly significant difference between the aqueous and organic extracts was observed, and that the organic extracts have high reducing power, especially the diethyl ether extract with an iron reduction value of 356.7 ± 0.92 *µ*g AAE/mg E.

#### 3.3.3. Antibacterial Activity

According to our bibliographic research, this is the first study dedicated to the evaluation of the antibacterial activity carried out on the *L. comosa* bulbs. The results of the antibacterial activity were expressed from the measurement of the diameter of the inhibition halos. According to Table 4, extracts of *L. comosa* showed activity against *Proteus mirabilis* with an inhibition diameter ranging from 8.5 mm for the ethanolic extract to 10.5 mm for diethyl ether extract at the concentration of 100 mg/mL, while for *Listeria innocua* only the hot and cold prepared diethyl ether extracts showed an inhibition zone ranging from 9 mm to 10 mm.

### 3.4. Principal Component Analysis (PCA)

In our study, the PCA was performed on individuals represented by the different extracts prepared from *L. comosa* bulbs and the variables are the measurements concerning the dosage of polyphenols, flavonoids, and tannins, the five tests of antioxidant activity (H_2_O_2_, ABTS, DPPH, FRAP, and RP), and the three tests of antidiabetic activity**:** alpha-amylase, alpha-glucosidase, and beta-galactosidase inhibition.

#### 3.4.1. Correlation Matrix (Pearson (*n*))

The correlation matrix between the methods of antioxidant activity and antidiabetic activity and the results of the determination of polyphenols, flavonoids, and tannins are shown in Table 5. According to the latter, we noticed a better correlation between polyphenols and flavonoids with a correlation coefficient *r* which is equal to 0.9705 and between flavonoids and tannins with *r* = 0.8396. For the antioxidant activity tests, we found that there is a difference in correlation between the five tests, with the best correlations found for the DPPH test with the FRAP test with a correlation coefficient (*r* = 0.9439), DPPH, and RP (*r* = 0.9549) and between DPPH and ABTS (*r* = 0.8982). The H_2_O_2_ test showed a low correlation with the ABTS test (*r* = 0.4240) and with the DPPH test (*r* = 0.5792). A positive correlation was observed for the content of polyphenols and flavonoids, with the ABTS, RP, and FRAP tests. The H_2_O_2_ test showed a positive correlation with the tannin content of the extracts (*r* = 0.8354). For the antidiabetic activity tests, we noticed that there is a weak correlation between the three tests alpha-amylase, alpha-glucosidase, and beta-galactosidase.

#### 3.4.2. Graphical Representation of the Principal Component Analysis (PCA)

**Figure 1:**
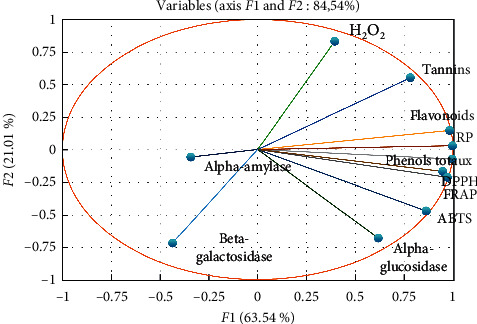
Graphical representation of the principal component analysis (PCA) of the different variables of the chemical composition and tests for antioxidant and antidiabetic activities. H_2_O_2_: hydrogen peroxide scavenging assay; ABTS: Trolox equivalent antioxidant capacity (TEAC) method/ABTS radical cation decolorization assay; FRAP: ferric reducing-antioxidant power assay; RP: reducing power method; DPPH: DPPH scavenging activity.

The results of the PCA are presented according to the two axes *F*1 and *F*2 because their cumulative percentage explains 84.54% of the information retained (Figure 1). The first principal component (*F*1) explains 63.54% of the total information, and the second one (*F*2) shows 21.01%.

According to Figure 1, the F1 axis is mainly constructed by the positive correlation between the ABTS, DPPH, RP, and FRAP tests and the contents of polyphenols, flavonoids, and tannins. The F2 axis is formed by the H_2_O_2_, alpha-glucosidase, and beta-galactosidase inhibition assays (Figure 1).

## 4. Discussion

### 4.1. Mineral Composition of *Leopoldia comosa* (L.) Bulbs

According to our bibliographical research, the present study represents the first investigation carried out on the mineral composition of *L. comosa* bulbs. The results obtained showed that *L. comosa* is an important source of the mineral elements: Fe, K, P, Na, Cu, Mg, and Ca, which are involved in the defense mechanism against oxidative stress and thus protect the body from cancer and cardiovascular disease [30]. Among the best-known antioxidant minerals such as zinc and selenium, zinc plays a global antioxidant role, as it is directly involved in the constitution of an anti-free radical enzyme: superoxide dismutase. This mineral is involved in the activity of more than 200 enzymes, particularly those involved in protection against free radicals and those involved in protein synthesis. Hence, it is important in the phenomena of cell renewal, cicatrization, and immunity. Selenium also participates in the fight against free radicals, being an essential component of certain antioxidant enzymes. It also has a stimulating effect on immunity and therefore contributes in general to the body's defense reactions.

The bulb of *L. comosa* could be considered as a good dietary complement because of its high content of Fe, K, P, Cu, and Mg, and because of its low Na/K ratio, it could also be used as a protective agent against cardiovascular diseases.

The food use of the bulb of *L. comosa* has a long history in Mediterranean countries. Indeed, the shape and taste of the *L. comosa* bulb are very similar to garlic, onion, and leek. In the past, peasants, during their work, picked and ate the bulbs with bread. Nowadays, the bulbs are peeled, cut, and fried in olive oil, sometimes mixed with cheese and eggs. In some places, for example, in the region of Salerno, Italy, they are boiled and served with a sweet and sour sauce [31]. *L. comosa* bulbs contain mucilages, sugars, latex, waxes, and traces of volatile oil. Thanks to these substances, the plant is widely used in food [31].

### 4.2. Yields of Aqueous and Organic Extractions of *Leopoldia comosa* (L.) Bulbs

The solvents used for *L. comosa* extraction showed significantly different extraction capacities between aqueous and organic extracts. From the results, we note that the extraction yield depends on the choice of solvent, extraction time, temperature, and extraction modality. Other studies have reported that the yield also varies according to the chemical nature of the sample [32]. In our study, the more polar the solvent, the higher and more important the yield is, which is consistent with the results of other work carried out by our laboratory on plants of the Taza region [10, 13–15]. Also, hot extraction by decoction modality seems to be the best method to obtain a better aqueous extraction yield, which agrees with the results of our laboratory by Bouabid et al. [10], who stated that the decoction of *Atractylis gummifera* (L.) gives a yield of 35% against 24% for maceration. A similar result was obtained in our work on another plant *Juglans regia* (L.) of the family Juglandaceae [13]. Indeed, the decoction of *Juglans regia* (L.) gives a yield of 14% compared to 8.75% for the aqueous macerate. The results of our study concur with those of previous studies in our laboratory which have shown that the extraction yield depends on the choice of solvent and extraction method [10, 13–15] and that for a better yield. The use of polar solvents is recommended.

In comparison with other work carried out on *L. comosa* bulbs, the study carried out by Larocca et al. in Italy reported a yield of 7.61 ± 0.10% for the extract prepared by the hydroalcoholic solvent (water/methanol 70%) by maceration using centrifugation of 80 rpm (rotation per minute) for 24 hours at 30°C [33]. Another study conducted by Loizzo et al. [34] in Italy on *L. comosa* bulbs showed that the yield was 3.16% for the ethanolic extract prepared by maceration for 48 hours compared to 1.3% for the macerated ethanolic extract of Moroccan *L. comosa* bulbs. So, the yield is almost twice what we got in our study. This can be explained by the geographical place of the plant's harvest, as well as the application of centrifugation in the maceration process.

The results of the hydrodistillation method from the bulbs of *L. comosa* showed us an absence of essential oils in this plant part, as according to our bibliographical research, no previous report has described the presence of essential oils in *L. comosa* bulbs. Other methods can be used to confirm the absence of essential oils in *L. comosa* bulbs, such as steam extraction, organic solvent extraction, or ultrasonic extraction.

### 4.3. Phytochemical Screening

The results of the phytochemical screening revealed the presence of flavonoids, catechin tannins, and quinones in the plant bulb and the 9 aqueous and organic extracts prepared. For the families of anthracenosides and anthraquinones, we can deduce that distilled water does not allow for their extraction and that only organic extracts allow for the extraction of these two families. Tests for alkaloids, saponins, and sterols were negative on the bulb and all prepared extracts.

In addition to the presence of phenolic compounds in *L. comosa* bulbs, previous studies have reported the presence of other chemical families. Indeed, Parrilli et al. [35] reported that the bulb of *L. comosa* is an important source of triterpene and glycoside, of which eucosterol 4a, a terpene of the nor-27 lanostane family, is the major compound. The same team was able to characterize the structure of glycosides from *L. comosa* [36]. In 1984, Adinolfi et al. were able to determine the structure of new triterpenes from *L. comosa* bulbs harvested in Italy [37, 38]. These authors continued their research and were able to identify two new 3-benzyl-chromanones, named 7-O-methyl-3.9-dihydropunctatin 1 and 8-O-demethyl-7-O-methyl-3.9-dihydropunctatin 2 [39], and in 1985, they were able to isolate, from the bulb of *L. comosa* harvested in Italy, three new homoisoflavanones, and their structures were elucidated: muscomosin, comosin, and 8-odemethyl-8-O-acetyl-7-O-methyl-3.9-dihydropunctatin [40].

A comparison with other plants from the Taza region studied under the same experimental conditions in our laboratory (SNAMOPEQ) allows us to say that the presence or absence of different secondary metabolites varies according to the botanical family, the species, and the geographical place of the plant's harvest as well as the different solvents used for extraction. For example, the study carried out by Senhaji et al. [15] showed that *Anabasis aretioïdes* harvested in the Figuig region is characterized by the presence of saponins, catechin tannins, and sterols, whereas the qualitative study carried out by Bentabet et al. on the same plant harvested in Algeria shows the presence of alkaloids, tannins, saponins, reducing sugars, and coumarins [41]. Another study was carried out by Senhaji et al. [14] on *Ajuga iva* Subsp. *Pseudoiva* showed the presence of 4 chemical families, flavonoids, sterols, saponins, and catechin tannins, while another study review reported the presence of other families for the plant *Ajuga iva* [42]. The study conducted by Bouabid et al. [10] on *Atractylis gummifera* (L.) which belongs to the Asteraceae family revealed that the plant contains flavonoids, tannins, saponins, quinones, and sterols. The families of Asteraceae (*Atractylis gummifera*) and Lamiaceae (*Ajuga iva* Subsp. *Pseudoiva*) are among the most exploited families in traditional Moroccan medicine [43, 44].

### 4.4. Polyphenol, Flavonoid, and Tannin Contents of *Leopoldia comosa* Bulbs

The contents of polyphenols, flavonoids, and tannins in an extract are parameters that depend strongly on the operating conditions of the extraction, and in particular on the nature and polarity of the solvent [12–15]. As Table 1 shows, the solvents used in extraction affected significantly (*P* < 0.05) the content of phenolic compounds in *L. comosa.*

The solvents diethyl ether followed by acetone and ethanol were found to be more effective in extracting phenolic compounds than water. This result indicates that *L. comosa* bulbs contain many fewer polar compounds.

Although water extraction gives us a high extraction yield, it is not the right solvent for the extraction of phenolic compounds. This could be explained by the fact that water extracts only water-soluble bioactive compounds; besides, many other residual substances and impurities are present in aqueous extracts. Organic extracts have higher levels of phenolic compounds than aqueous extracts.

The results obtained are in agreement with many results previously reported by our laboratory indicating that phenolic compounds are generally more soluble in polar organic solvents than in water [12, 13, 15].

In comparison with the work carried out by Casacchia et al. on the bulb *L. comosa* from Italy [45], the contents of phenolic compounds were of the order of 39.53 ± 0.027 and 49.80 ± 0.012 mg CAE/g MF (chlorogenic acid equivalent (CAE) per g fresh material (MF)), respectively, for the decocted and the steamed bulb extract cooked for 15 min; these results are expressed by another reference standard (chlorogenic acid) and not the gallic acid which we used and which is the most used. For the flavonoid content, the decocted has a value of 0.64 ± 0.026 and the steamed bulb extract of 1.63 ± 0.010 mg QE/g MF (mg equivalent of quercetin per gram of fresh material). Similarly, the study carried out by Larocca et al. in Italy [33] reported the content of phenolic compounds and flavonoids for the hydroalcoholic extract (water/methanol 70%) which was around 57.67 ± 0.72 mg GAE/g E and 18.79 ± 0.36 mg QE/g E, respectively.

According to our bibliographic research, the tannin content of *L. comosa* bulbs has not been reported in any previous reports, and our study is the first one that has investigated and dosed the tannins.

Our study has highlighted the presence and content of tannins for the first time for the bulb of *L. comosa*, so we can say that Moroccan *L. comosa* has high contents of polyphenols, flavonoids, and in particular tannins extracted by the less polar solvent diethyl ether. Also, we can deduce that the choice of the solvents to be used is essential for the extraction and determination of the secondary metabolites of a plant and its pharmacological valorization by the studies of the biological properties.

### 4.5. Antidiabetic Activity

In several epidemiological studies, postprandial glycemia is a major independent risk factor for cardiovascular disease in both glucose intolerant and type 2 diabetic patients. People with low glucose tolerance or diabetes often have high postprandial blood glucose levels for long periods [46, 47]. Alpha-amylase and alpha-glucosidase are two enzymes responsible for the degradation of carbohydrates. This degradation allows the absorption of glucose and increases blood glucose levels [10, 48]. As a result, inhibition of these two enzymes limits the increase in postprandial glycemia and may therefore be an important strategy for reducing blood glucose levels in type 2 diabetics.

The results of the present study reveal that the aqueous and organic extracts of *L. comosa* bulbs have a high alpha-amylase inhibition activity, with a high inhibitory power of IC50 of 2752.33 ± 8.11 *µ*g/mL for the decocted and an IC50 of 2264 ± 22.86 *µ*g/mL for the ethanolic extract. In comparison with other studies on the bulb of *L. comosa*, Casacchia et al. in Italy reported IC50s of 730 ± 0.13 *µ*g/mL and 690 ± 0.02 *µ*g/mL for the extract of the bulb steamed for 15 min and decocted, respectively [45]. In another study carried out in Italy on *L. comosa* bulbs, Larocca et al. found an IC50 of 75.17 ± 0.52 *µ*g/mL for the hydroalcoholic extract (water/methanol 70%) [33]. Similarly, the study by Loizzo et al. in the same country found IC50s of 81.3 ± 2.77 and 166.9 ± 3.4 *µ*g/mL for ethanolic and n-hexane extracts prepared from the bulb of *L. comosa*, respectively [34]. Therefore, we can conclude that the results of the alpha-amylase inhibition activity of *L. comosa* bulb extracts are different even for bulbs harvested in the same country, which can be explained by several parameters, including the choice of solvent, extraction ratio, extraction method, treatment or not of the plant before use, and place and season of harvest of the plant.

The results of alpha-glucosidase inhibition by aqueous and organic extracts of *L. comosa* bulbs are promising, mainly the acetone extract (IC50 = 85.41 ± 3.86 *µ*g/mL) which proved to be 3 times more active than the reference standard, acarbose (IC50 = 247.23 ± 2.85 *µ*g/mL). Our results are consistent with the work carried out by Larocca et al. in Italy who reported an IC50 of 85.33 ± 0.38 *µ*g/mL for the hydroalcoholic extract (water/methanol 70%) of *L. comosa* bulbs [33]. However, our results are better than those obtained by Loizzo et al. who reported IC50s of 112.8 ± 3.3 and 166.9 ± 3.4 *µ*g/mL, respectively, for the ethanolic and n-hexane extract of *L. comosa* bulbs [34].

The results of inhibition of aqueous and organic extracts of *L. comosa* bulbs by the beta-galactosidase inhibition test are presented for the first time in our study. All the extracts tested showed a high hypoglycemic power with a better activity presented by the aqueous extracts, especially the decocted (IC50 = 205.43 ± 2.22 *µ*g/mL), and for the organic extracts, the acetone extract was the most active with an IC50 value of 163.5 ± 2.51 *µ*g/mL.

### 4.6. Antiradical and Antioxidant Activity

#### 4.6.1. Hydrogen Peroxide Scavenging Assay (H_2_O_2_)

The study of the antioxidant activity of *L. comosa* bulbs by the H_2_O_2_ test represents the first study carried out by this test. The importance of this test is shown by the ability of the extracts tested to scavenge the H_2_O_2_ radical, as the latter is an oxidant and can directly inactivate some enzymes, generally by oxidation of essential thiol groups (-SH). Hydrogen peroxide can rapidly cross the cell membrane and once inside the cell, H_2_O_2_ can probably react with Fe^2+^ and eventually Cu^2+^ and form a hydroxyl radical, and this may be the cause of its many toxic effects. Therefore, the removal of hydrogen peroxide is very important. The aqueous and organic extracts of *L. comosa* were tested for their antioxidant capacity by the hydrogen peroxide scavenging method, and as shown in Table 3, all extracts, at the concentration of 100 *µ*g/mL, showed scavenging capacity against H_2_O_2_. In our study, diethyl ether extract represents the highest percentage of H_2_O_2_ scavenging (62.67 ± 0.06%), which is significantly higher than that obtained by aqueous and other organic extracts. These results are higher than those obtained in our laboratory [12, 14, 15] who found that the macerated methanolic extract of *Atractylis gummifera* (L.), *Ajuga iva* Subsp. *Pseudoiva*, and *Anabasis aretioïdes* gives a percentage of scavenging, respectively, of the order of 19.24 ± 1.10%, 22.17 ± 0.30%, and 5.32 ± 0.23%.

#### 4.6.2. Trolox Equivalent Antioxidant Capacity Using ABTS (TEAC)

The results of the ABTS test represent the first study conducted using this test because according to our literature search, no studies have been done using this test for the bulb of *L. comosa*. In this test, the antioxidant reduces the ABTS•+ cation radical generated by ammonium persulfate. Our results show that aqueous extracts have lower TEAC contents than organic extracts and that diethyl ether extract gives the highest TEAC value (381.63 ± 0.63 *µ*g TE/mg E) which is highly correlated with the content of polyphenols, flavonoids, and tannins in this extract. Extraction by organic solvents using Soxhlet in our study seems to be the most efficient method to extract phenolic compounds and therefore allows obtaining a very good antioxidant activity. These results disagree with previous work carried out by our laboratory which indicates that organic extraction by maceration is the best method to obtain good antioxidant activity [12–15] which can be explained by the qualitative and quantitative difference in the chemical composition of our plant subject of this study.

#### 4.6.3. 2, 2-Diphenyl-1-picrylhydrazil Free Radical Scavenging Activity (DPPH)

The DPPH radical scavenging test showed an activity that is dependent on the nature of the solvent used, the extraction modality, and the concentration tested. Thus, the diethyl ether extract of *L. comosa* bulbs at 5 *µ*g/mL showed a scavenging effect of 31.10% which increases to 96.43% at 100 *µ*g/mL.

IC50 values were calculated for all aqueous and organic extracts and reference standards (ascorbic acid, Trolox, and BHT); a lower IC50 value indicates higher antiradical activity. For the aqueous extracts, we note that the decocted presents the best activity with an IC50 of 1011.33 ± 4.37 *µ*g/mL. Organic extracts showed higher activity compared to aqueous extracts with an IC50 of 10.08 ± 0.01 *µ*g/mL for the diethyl ether extract. Therefore, the polar solvent diethyl ether is the most suitable solvent for *L. comosa* to extract its bioactive molecules. Our results are better in comparison with previous work carried out in Italy by Loizzo et al. [34] on the macerated ethanolic extract and the hexane extract of *L. comosa* which obtained IC50 values of the order of 40.9 ± 1.8 and 46.6 ± 1.5 *µ*g/mL, respectively. Another study carried out by Larocca et al. in Italy found an IC50 of 36.73 ± 0.49 *µ*g/mL for the hydroalcoholic extract (70% water and methanol) [33]. Similarly, for the study carried out by Casacchia et al. in Italy, the decoction recorded an IC50 value of 9630 *µ*g/ml [45]. These results can be explained by the choice of extraction solvents and by the hot and cold extraction modality adopted and also by the chemotype.

#### 4.6.4. Ferric Reducing-Antioxidant Power Assay (FRAP)

The reduction and oxidation of a chemical are defined as a gain or loss of electrons, respectively. A reducing agent is a substance that gives electrons and, therefore, causes the reduction of another reagent. The FRAP test is widely used in the evaluation of the antioxidant power of food polyphenols. The antioxidant potency of *L. comosa* extracts was estimated based on its ability to reduce the TPTZ-Fe (III) complex to TPTZ-Fe (II) measured at wavelength 593 nm. It appears that organic extracts show a higher antioxidant capacity by the FRAP test than aqueous extracts. The reducing capacity of the diethyl ether extract was the most powerful among the nine extracts tested (394.77 ± 0.74 *µ*g TE/mg E). These results correlate with work carried out in our laboratory [13–15] which found that organic extracts show great antioxidant power compared to aqueous extracts and that hot extraction gives better results than cold extraction by this test.

#### 4.6.5. Reducing Power Assay (RP)

The reducing power test is based on the reduction of the Fe^3+/^ferricyanide complex to ferrous ion (Fe^2+)^ in the presence of reducing agents (antioxidants) measured at wavelength 700 nm. In this test, the classification of the extracts is the same as that obtained with the FRAP test, and this means that the molecules responsible for the reduction of the complex are the same as those of the reducing power. The diethyl ether extract proved to be the most active with an iron reduction value of 356.7 ± 0.92 *µ*g AAE/mg E which is due to its high content of phenolic compounds and therefore its ability to transfer electrons. The study conducted by Loizzo et al. in Italy [34] reported that the ethanolic extract prepared by cold maceration for 48 hours and the n-hexane extract had IC50s of 78.8 ± 2.8 and 113.6 ± 3.7 *µ*g/mL, respectively.

### 4.7. Antibacterial Activity

On this plant and to the best of our knowledge, this study is the first to provide data on antibacterial activity. The results show that among all the extracts tested, the diethyl ether extract has a moderate antibacterial effect on two strains: one Gram- (*Listeria innocua*) and the other Gram+ (*Proteus mirabilis*). The promising effect of the diethyl ether extract could be attributed to its phenolic content with values, respectively, of the order of 129.75 ± 0.29 *µ*g GAE/mg E, 988.26 ± 0.18 *µ*g QE/mg E, and 30.22 ± 0.15 *µ*g CE/mg E for polyphenols, flavonoids, and tannins. Indeed, phenolic compounds have been shown to have strong antibacterial activity [49].

Using the same bacterial strains, the work carried out in our laboratory (SNAMOPEQ) with organic extracts (methanol, macerated methanol, chloroform, ethyl acetate, and petroleum ether) prepared from the aerial part of *Anabasis aretioïdes* showed that the inhibition diameter varies from 7 to 13.5 mm depending on the bacterial strain used and the choice of solvent for extraction. Of the five extracts tested, the ethyl acetate extract at a concentration of 100 mg/mL showed moderate antibacterial activity against *Staphylococcus aureus* CECT976, *Proteus mirabilis*, *Bacillus subtilis* DSM6633, *Escherichia coli* K12, and *Pseudomonas aeruginosa* CECT118 with an inhibition diameter of 13.5, 12.5, 11.5, 10.5, and 8 mm, respectively. However, the less polar extract prepared by petroleum ether did not affect the bacterial strains [15]. These results are different from our study which revealed that the diethyl ether extract whose polarity is close to that of the petroleum ether was found the most active. Therefore, the use of different solvents for extraction is important to select the one that gives the best result.

Besides, the antibacterial activity of phenolic compounds has been extensively studied against a wide range of microorganisms and has demonstrated potent activity and interesting synergistic properties with antibiotics [49, 50]. However, the absence of activity for some extracts of *L. comosa* does not mean the total absence of phenolic compounds but could be due to the low amount of these compounds or their antagonistic action by the presence of other compounds. The compounds responsible for the antibacterial activity of the *L. comosa* bulb are characterized by their solubility in less polar solvents.

### 4.8. Principal Component Analysis (PCA)

PCA was performed with *n* = 9 extracts prepared from *L. comosa* bulbs. The PCA is associated with a diagonal Pearson correlation matrix between the nine extracts and the principal component factors. The results of the present study allowed us to determine the different correlations between polyphenols, flavonoids, and tannins; the five methods of antioxidant activity; and the three tests of antidiabetic activity. According to Figure 1, we found a positive correlation between the ABTS, DPPH, RP, and FRAP tests and the contents of polyphenols, flavonoids, and tannins. These results are in agreement with the literature which indicates that phenolic compounds play an important role in the scavenging of free radicals [13, 51, 52]. For tests of antidiabetic activity, we noticed a weak correlation between the three tests alpha-amylase, alpha-glucosidase, and beta-galactosidase and the contents of polyphenols, flavonoids, and tannins, which is in disagreement with the literature of which several studies have reported that polyphenols can have considerable hypoglycemic properties [12, 46]. This can be explained by the antagonistic action between the families present in *L. comosa* bulbs or the presence of other chemical families in the bulbs of *L. comosa* that we have not dosed and which have a greater action in the inhibition of the enzymes responsible for the antidiabetic activity.

## 5. Conclusions

This study, undertaken for the first time in Morocco, allowed us to describe and determine the mineral, chemical composition, and pharmacological properties of the aqueous and organic extracts of the bulb of *Leopoldia comosa* (L.), a spontaneous plant from the region of Taza, Morocco, which is characterized by a high production of this plant.

*L. comosa* is an important source of the mineral elements in particular: Fe (33552), K (1843.14), P (756.36), Na (439.65), Cu (303.9), Mg (272.37), and Ca (20.55) mg/kg plant matter.

Phytochemical screening carried out both on the bulb and the aqueous and organic extracts prepared from it shows that *L. comosa* is rich in polyphenol, flavonoid, tannin, quinone, anthraquinone, and anthracenoside compounds, mainly the diethyl ether extract. The results of the *in vitro* antidiabetic activity showed that *L. comosa* extracts possess inhibitory activity of the enzymes alpha-amylase, alpha-glucosidase, and beta-galactosidase, in particular, the aqueous extract prepared by decoction and the acetone extract, which were found to be the most active in all three tests. The results of the antioxidant activity show that all the extracts prepared from *L. comosa* have high antioxidant power, especially the diethyl ether extract prepared by Soxhlet, which presents significant values via the five tests of antioxidant activity (H_2_O_2_, ABTS, DPPH, FRAP, and RP). The results of the antibacterial activity show that among all the extracts tested, the diethyl ether extract has a moderate antibacterial effect on two strains: one Gram- (*Listeria innocua*) and the other Gram+ (*Proteus mirabilis*). The results of the principal component analysis (PCA) allowed us to conclude that there is a positive correlation between the ABTS, DPPH, RP, and FRAP tests and the contents of polyphenols, flavonoids, and tannins. For antidiabetic activity, a weak correlation was obtained between the three assays alpha-amylase, alpha-glucosidase, and beta-galactosidase and phenolic compounds, flavonoids, and tannins.

In addition to the economic and ethnomedicinal values represented by wild Moroccan *L. comosa* because it represents an important source of income for the population in the region of Taza, Morocco, and is used in traditional Moroccan medicine [16], the present study underlines the importance of *L. comosa* as a medicinal and food plant by highlighting its richness in mineral elements and chemical compounds at the origin of the important biological antidiabetic, antioxidant, and antibacterial activities. Our results of the mineralogical and chemical analyses and the *in vitro* evaluation of the plant's antidiabetic and antioxidant activities are promising and encourage us to continue the *in vivo* study of the antidiabetic and antioxidant activities mainly for the aqueous decocted extract and the organic acetone extract.

## Figures and Tables

**Table 1:** Polyphenol, flavonoid, and tannin contents of aqueous and organic extracts of *Leopoldia comosa* (L.) bulbs.

Extracts of *Leopoldia comosa* bulbs		Polyphenols (*µ*g GAE/mg E)^x^	Flavonoids (*µ*g QE/mg E)^y^	Tannins (*µ*g CE/mg E)^z^
Aqueous	Decocted	**4.28** **±** **0.02**^a^	82.15 ± 0.26^a^	17.06 ± 0.11^a^
	Infused	**4.44** **±** **0.02**^a^	**90.82** **±** **0.59**^b^	16.62 ± 0.04^b,a^
	Macerated	**4.69** **±** **0.01**^a^	78.63 ± 0.21^c, a^	**18.68** **±** **0.11**^c^

Organic	Ethanolic	20.49 ± 0.08^b^	147.63 ± 0.57^d^	12.28 ± 0.17^d^
	Macerated ethanolic	18.20 ± 0.04^c^	128.00 ± 0.23^e^	9.8 ± 0.18^e^
	Acetone	69.96 ± 0.01^d^	330.15 ± 1.45^f^	16.2 ± 0.23^f, a, b^
	Macerated acetone	61.43 ± 0.04^e^	308.45 ± 0.6^g^	15.06 ± 0.15^g^
	Diethyl ether	**129.75** **±** **0.29**^f^	**988.26** **±** **0.18**^h^	**30.22** **±** **0.15**^h^
	Macerated diethyl ether	115.81 ± 0.24^g^	793.67 ± 1.49^i^	23.24 ± 0.09^i^

Data are expressed as mean ± standard deviation (*n* = 3). Different letters in the same column indicate a significant difference (*P* < 0.05). ^x^*µ*g of gallic acid equivalent per mg of dry plant extract. ^y^*µ*g of quercetin equivalent per mg of dry plant extract. ^z^*µ*g of catechin equivalent per mg of dry plant extract.

**Table 2:** IC50 (*µ*g/mL) of aqueous and organic extracts of *Leopoldia comosa* (L.) bulbs for the inhibition of alpha-amylase, alpha-glucosidase, and beta-galactosidase assays.

Extracts of *Leopoldia comosa* bulbs		Alpha-amylase (IC50 *µ*g/mL)^*x*^	Alpha-glucosidase (IC50 *µ*g/mL)^*x*^	Beta-galactosidase (IC50 *µ*g/mL)^*x*^
Aqueous	Decocted	**1200.66** **±** **13.79**^a^	**238.53** **±** **2.35**^a^	**216.9** **±** **8.67**^a^
	Infused	2880 ± 8.05^b^	258.93 ± 1.38^b^	**205.43** **±** **2.22**^b^
	Macerated	2752.33 ± 8.11^c^	268.23 ± 2.85^c, b^	245.5 ± 9.26^c^

Organic	Ethanolic	2264 ± 22.86^d^	257.96 ± 2.72^d,b,c^	182.23 ± 7.88^d^
	Macerated ethanolic	2384 ± 7.40^e^	162.7 ± 2.79^e^	196.2 ± 4.42^e^
	Acetone	**2219.33** **±** **3.31**^f,d^	**85.41** **±** **3.86**^f^	**163.5** **±** **2.51**^f^
	Macerated acetone	2289.66 ± 7.45^g^^,d^	**85.95** **±** **1.92**^g^^,f^	200.43 ± 12.15^g^
	Diethyl ether	2512.33 ± 5.98^h^	136.03 ± 0.95^h^	240.23 ± 13.45^h^
	Macerated diethyl ether	2897.66 ± 4.76^i, b^	130.80 ± 1.39^i, h^	291.83 ± 10.83^i^

Reference standard	Acarbose	616.33 ± 6.58^j^	**195 ± 5** ^j^	—
	Quercetin	—	—	171.16 ± 2.90^j^

Data are expressed as mean ± standard deviation (*n* = 3). Different letters in the same column indicate a significant difference (*P* < 0.05). ^**x**^Concentration that inhibits 50% of the activity in micrograms per milliliter.

**Table 3:** Antiradical and antioxidant activity of aqueous and organic extracts of *Leopoldia comosa* (L.) bulbs via the five tests H_2_O_2_, ABTS, DPPH, FRAP, and RP.

Extracts of *Leopoldia comosa* bulbs		H_2_O_2_ (%)^*a*^	ABTS (*µ*g TE/mg E)^*b*^	DPPH (IC50 *µ*g/mL)^c^	FRAP (*µ*g TE/mg E)^*b*^	RP (*µ*g AAE/mg E)^*d*^
Aqueous	Decocted	**62.12** **±** **0.2**^***a*, *d***^	**27.46** **±** **0.69**^***a***^	**1011.33** **±** **4.37**^***a***^	12.9 ± 0.1^*a*^	**8.36** **±** **0.06**^***a***^
	Infused	61.89 ± 0.3^*a*, *d*, *e*^	17.18 ± 0.17^*b*^	1089.33 ± 0.92^*b*^	11.16 ± 0.52^*b*, *a*^	**7.91** **±** **0.14**^***a***^
	Macerated	61.72 ± 0.1^*a*, *d*, *e*^	6.63 ± 0.31^*c*^	1140 ± 20.64^*c*^	**15.27** **±** **0.1**^***c***^	**10.68** **±** **0.13**^***a***^

Organic	Ethanolic	61.3 ± 0.16^*b*, *a*^	225.86 ± 1.04^*d*^	139.4 ± 6.93^*d*^	131.55 ± 0.26^*d*^	59.40 ± 0.21^*b*^
	Macerated ethanolic	61.09 ± 0.05^*c*, *b*^	89.47 ± 0.68^*e*^	220.5 ± 2.91^*e*^	49.24 ± 0.13^*e*^	18.86 ± 0.05^*c*^
	Acetone	61.24 ± 0.2^*d*, *b*, *c*^	364.96 ± 0.28^*f*^	99.76 ± 0.04^*f*, *d*^	277.74 ± 0.67^*f*^	147.39 ± 1.07^*d*^
	Macerated acetone	60.94 ± 0.2^*e*, *b*, *c*^	343.02 ± 1.44^*g*^	100 ± 0.03^*g*^^, *f*, *d*^	225.77 ± 0.15^*g*^	133.32 ± 0.8^*e*^
	Diethyl ether	**62.67** **±** **0.06**^***f***^	**381.63** **±** **0.63**^***h***^	**10.08** **±** **0.01**^***h***^	**394.77** **±** **0.74**^***h***^	**356.7** **±** **0.92**^***f***^
	Macerated diethyl ether	61.91 ± 0.1^*g*^^, *a*^	360.93 ± 0.25^*i*, *f*^	10.15 ± 0.04^*i*, *h*^	358.77 ± 0.74^*i*^	283.95 ± 0.59^*g*^

Reference standards	Trolox	—	—	1.75 ± 0.09j	—	—
	Ascrobic acid	**63.63** **±** **0.47**^***f***^	—	0.17 ± 0.02k	—	—
	*B*HT	—	—	0.17 ± 0.02k	—	—

Data are expressed as mean ± standard deviation (*n* = 3). Different letters in the same column indicate a significant difference (*p* < 0.05). ^***a***^H_2_O_2_ scavenging activity (%) of *L. comosa* extracts at the concentration of 100 *μ*g/mL. ^***b***^*μ*g of Trolox equivalent per mg of dry plant extract. ^***c***^Concentration that inhibits 50% of the activity in micrograms per milliliter. ^***d***^*μ*g of ascorbic acid equivalent per mg of dry plant extract.

**Table 4:** Diameters of the inhibition zone (in mm) of different organic extracts from *Leopoldia comosa* (L.) bulbs against six pathogenic bacterial strains.

Strains	Ethanolic extract			Macerated ethanolic extract			Acetone extract			Macerated acetone extract			Diethyl ether extract			Macerated diethyl ether extract			Standard (+)		Standard (−)
	40 mg/ml	80 mg/ml	100 mg/ml	40 mg/ml	80 mg/ml	100 mg/ml	40 mg/ml	80 mg/ml	100 mg/ml	40 mg/ml	80 mg/ml	100 mg/ml	40 mg/ml	80 mg/ml	100 mg/ml	40 mg/ml	80 mg/ml	100 mg/ml	Tetra 20 *µ*g/ml	AK 30 *µ*g/ml	DMSO (10%)
B.G-																					
*Ecoli*	—	—	—	—	—	—	—	—	—	—	—	—	—	—	—	—	—	—	12	0	0
*Prote*	7	7	**8.5**	8	7	**7.5**	9	7.5	**8**	10	7	**9.5**	11	8	**10.5**	10	10	**10.5**	23	0	0
*Psdm*	—	—	—	—	—	—	—	—	—	—	—	—	—	—	—	—	—	—	0	21	0
B.G+																					
*Staph*	—	—	—	—	—	—	—	—	—	—	—	—	—	—	—	—	—	—	13	0	0
*Bacil*	—	—	—	—	—	—	—	—	—	—	—	—	—	—	—	—	—	—	0	24.5	0
*Lister*	—	—	—	—	—	—	—	—	—	—	—	—	—	9	**9**	—	10	**10**	0	26.5	0

B.G-: Gram-negative bacteria; *Ecoli*: *Escherichia coli* K12; *Prote*: *Proteus mirabilis*; *Psdm*: *Pseudomonas aeruginosa* CECT118; B.G*+*: Gram-positive bacteria; *Staph*: *Staphylococcus aureus* CECT976; *Bacil*: *Bacillus subtilis* DSM6633; *Lister*: *Listeria innocua* CECT4030; Tetra: tetracycline; Ak: amikacin; DMSO: dimethylsulfoxide.

**Table 5:** Correlation coefficient between chemical composition and tests for antidiabetic and antioxidant activities of aqueous and organic extracts of *Leopoldia comosa* (L.) bulbs.

Variables	Polyphenols	Flavonoids	Tannins	H_2_O_2_	ABTS	RP	FRAP	DPPH	Alpha-amylase	Alpha-glucosidase	Beta-galactosidase
Polyphenols	**1**	**0.9705**	0.7381	0.3407	**0.8873**	**0.9905**	**0.9807**	**0.9545**	−0.3081	0.6769	−0.3813
Flavonoids		**1**	**0.8396**	0.5272	0.7708	**0.9890**	**0.9188**	**0.9233**	−0.3112	0.4969	−0.5158
Tannins			**1**	**0.8354**	0.4240	0.8007	0.6492	0.5792	−0.2023	0.1585	−0.6514
H2O2				**1**	−0.0375	0.4389	0.2218	0.2049	0.0719	−0.3094	−0.5861
ABTS					**1**	0.8515	**0.9549**	**0.8982**	−0.2220	0.8135	−0.0031
RP						**1**	**0.9646**	**0.9439**	−0.3006	0.5834	−0.4245
FRAP							**1**	**0.9549**	−0.2872	0.7234	−0.2399
DPPH								**1**	−0.3231	0.6222	−0.3180
Alpha-amylase									**1**	−0.1038	0.3942
Alpha-glucosidase										**1**	0.1751
Beta-galactosidase											**1**

H_2_O_2_: hydrogen peroxide scavenging assay; ABTS: Trolox equivalent antioxidant capacity (TEAC) method/ABTS radical cation decolorization assay; FRAP: ferric reducing-antioxidant power assay; RP: reducing power method; DPPH: DPPH scavenging activity.

## Data Availability

The experimental data used to support the findings of this study are incorporated into the article.

## Abbreviations

*L. comosa*: *Leopoldia comosa*
ROS: Reactive oxygen species
H_2_O_2_: Hydrogen peroxide scavenging assay
TEAC or ABTS: Trolox equivalent antioxidant capacity method/ABTS radical cation decolorization assay
FRAP: Ferric reducing-antioxidant power assay
RP: Reducing power method
DPPH: 1.1-Diphenyl-2-picrylhydrazyl scavenging activity
PCA: Principal component analysis
ANOVA: Analysis of variance
IC50: Concentration that causes 50% inhibition
EO: Essential oils.

## References

[1] Valko M., Leibfritz D., Moncol J., Cronin M. T. D., Mazur M., Telser J. (2007). Free radicals and antioxidants in normal physiological functions and human disease. *The International Journal of Biochemistry & Cell Biology*.

[2] Magalhães L. M., Segundo M. A., Reis S., Lima J. L. F. C. (2008). Methodological aspects about *in vitro* evaluation of antioxidant properties. *Analytica Chimica Acta*.

[3] Dias V., Junn E., Mouradian M. M. (2013). The role of oxidative stress in Parkinson’s disease. *Journal of Parkinson’s Disease*.

[4] Mahomoodally M. F., Subratty A. H., Gurib-Fakim A., Choudhary M. I., Nahar Khan S. (2012). Traditional medicinal herbs and food plants have the potential to inhibit key carbohydrate hydrolyzing enzymes In vitro and reduce postprandial blood glucose peaks In vivo. *The Scientific World Journal*.

[5] Song Y., Manson J. E., Buring J. E., Sesso H. D., Liu S. (2005). Associations of dietary flavonoids with risk of type 2 diabetes. and markers of insulin resistance and systemic inflammation in women: a prospective study and cross-sectional analysis. *Journal of the American College of Nutrition*.

[6] Hokayem M., Bisbal C., Lambert K., Avignon A. (2012). Which place for antioxidants in the prevention of type 2 diabetes?. *Médecine des Maladies Métaboliques*.

[7] WHO (World Health Organization) (2016). *World Diabetes Report*.

[8] Ministry of Health (2021). *World Health Day: Together against Diabetes*.

[9] Fauci A. S., Touchette N. A., Folkers G. K. (2005). Emerging infectious diseases: a 10-year perspective from the National Institute of allergy and infectious diseases. *Emerging Infectious Diseases*.

[10] Bouabid K., Lamchouri F., Toufik H., Sayah K., Cherrah Y., Faouzi M. E. A. (2018). Phytochemical screening and *in vitro* evaluation of alpha amylase. alpha glucosidase and beta galactosidase inhibition by aqueous and organic *Atractylis gummifera* L. extracts. *Plant Science Today*.

[11] Bouabid K., Lamchouri F., Toufik H., Boulfia M., Senhaji S., Faouzi M. E. A. (2019). *In vivo* anti-diabetic effect of aqueous and methanolic macerated extracts of *Atractylis gummifera*. *Bangladesh Journal of Pharmacology*.

[12] Bouabid K., Lamchouri F., Toufik H., Faouzi M. E. A. (2020). Phytochemical investigation. *in vitro* and *in vivo* antioxidant properties of aqueous and organic extracts of toxic plant: *Atractylis gummifera* L. *Journal of Ethnopharmacology*.

[13] Boulfia M., Lamchouri F., Toufik H. (2020). Chemical analysis. phenolic content. and antioxidant activities of aqueous and organic Moroccan *Juglans regia* L. Bark extracts. *Current Bioactive Compounds*.

[14] Senhaji S., Lamchouri F., Bouabid K. (2020). Phenolic contents and antioxidant properties of aqueous and organic extracts of a Moroccan Ajuga iva subsp. pseudoiva. *Journal of Herbs. Spices & Medicinal Plants*.

[15] Senhaji S., Lamchouri F., Toufik H. (2020). .Phytochemical content. antibacterial and antioxidant potential of endemic plant Anabasis aretioïdes coss. & moq. (Chenopodiaceae). *BioMed Research International*.

[16] Boulfia M., Lamchouri F., Lachkar N., Khabbach A., Zalaghi A., Toufik H. (2021). Socio-economic value and ethnobotanical study of Moroccan wild *Leopoldia comosa* L. *Ethnobotany Research and Applications*.

[17] Arora M., Kiran B., Rani S., Rani A., Kaur B., Mittal N. (2008). Heavy metal accumulation in vegetables irrigated with water from different sources. *Food Chemistry*.

[18] Singelton V. L., Orthofer R., Lamuela R. M. (1999). Analyses of total phenols and other oxidation substances and antioxidants by means of Folin-Ciocalteu Reagent. *Methods Enzymology*.

[19] Dewanto X., Wu K., Adom K., Liu R. H. (2020). Thermal processing enhances the nutritional value of tomatoes by increasing total antioxidant activity. *Journal of Agricultural and Food Chemistry*.

[20] Julkunen-Tiitto R. (1985). Phenolic constituents in the leaves of northern willows: methods for the analysis of certain phenolics. *Journal of Agricultural and Food Chemistry*.

[21] Wickramaratne M. N., Punchihewa J. C., Wickramaratne D. B. M. (2016). *In-vitro* alpha amylase inhibitory activity of the leaf extracts of *Adenanthera pavonina*. *BMC Complementary and Alternative Medicine*.

[22] Lordan S., Smyth T. J., Soler-Vila A., Stanton C., Ross R. P. (2003). The &-amylase and & glucosidase inhibitory effects of Irish seaweed extracts. *Food Chemistry*.

[23] Ruch R. J., Cheng S.-j., Klaunig J. E. (1989). Prevention of cytotoxicity and inhibition of intercellular communication by antioxidant catechins isolated from Chinese Green Tea. *Carcinogenesis*.

[24] Re R., Pellegrini N., Proteggente A., Pannala A., Yang M., Rice-Evans C. (1999). Antioxidant activity applying an improved ABTS radical cation decolorization assay. *Free Radical Biology and Medicine*.

[25] Sharma O. P., Bhat T. K. (2009). DPPH antioxidant assay revisited. *Food Chemistry*.

[26] Benzie I. F. F., Strain J. J. (1996). The ferric reducing ability of plasma (FRAP) as a measure of “antioxidant power”: the FRAP assay. *Analytical Biochemistry*.

[27] Oyaizu M. (1986). Studies on products of browning reaction. Antioxidative activities of products of browning reaction prepared from glucosamine. *The Japanese Journal of Nutrition and Dietetics*.

[28] Sharififar F., Moshafi M. H., Mansouri S. H., Khodashenas M., Khoshnoodi M. (2007). *In vitro* evaluation of antibacterial and antioxidant activities of the essential oil and methanol extract of endemic *Zataria multiflora* Boiss. *Food Control*.

[29] Shahidi F., Chandrasekara A. (2013). Millet grain phenolics and their role in disease risk reduction and health promotion: a review. *Journal of Functional Foods*.

[30] Elbadrawy E., Sello A. (2016). Evaluation of nutritional value and antioxidant activity of tomato peel extracts. *Arabian Journal of Chemistry*.

[31] Casoria P., Menale B., Muoio R. (1999). *Muscari comosum. Liliaceae*. in the food habits of south Italy. *Economic Botany*.

[32] El-Chaghaby G. A., Ahmad A. F., Ramis E. S. (2014). Evaluation of the antioxidant and antibacterial properties of various solvents extracts of *Annona squamosa* L. leaves. *Arabian Journal of Chemistry*.

[33] Larocca M., Di Marsico M., Riccio P., Rossano R. (2018). The *in vitro* antioxidant properties of *Muscari comosum* bulbs and their inhibitory activity on enzymes involved in inflammation. post-prandial hyperglycemia. and cognitive/neuromuscular functions. *Journal Food. Biochemistry*.

[34] Loizzo M. R., Tundis R., Menichini F. (2010). Antioxidant and hypoglycaemic potential ofMuscari comosum (L.) Mill. bulb extracts. *International Journal of Food Sciences and Nutrition*.

[35] Parrilli M., Lanzetta R., Dovinola V., Adinolfi M., Mangoni L. (1981). Glycosides from Muscari comosum. 1. Eucosterol glycoside and structure of its methanolysis products. *Canadian Journal of Chemistry*.

[36] Adinolfi M., Barone G., Lanzetta R., Laonigro G., Mangoni L., Parrilli M. (1983). Glycosides from muscaricomosum. 4. Structure of muscaroside A. *Canadian Journal of Chemistry*.

[37] Adinolfi M., Barone G., Lanzetta R., Laonigro G., Mangoni L., Parrilli M. (1984). Triterpenes from bulbs of *Muscari comosum*. 2. The structure of two novel nortriterpenes. *Journal of Natural Products*.

[38] Adinolfi M., Barone G., Lanzetta R., Laonigro G., Mangoni L., Parrilli M. (1984). Triterpenes from bulbs of *Muscari comosum*. 4. The structure of further novel nortriterpene components. *Journal of Natural Products*.

[39] Adinolfi M., Barone G., Belardini M., Lanzetta R., Laonigro G., Parrilli M. (1984). 3-Benzyl-4-chromanones from Muscari comosum. *Phytochemistry*.

[40] Adinolfi M., Barone G., Belardini M., Lanzetta R., Laonigro G., Parrilli M. (1985). Homoisoflavanones from *Muscari comosum* bulbs. *Phytochemistry*.

[41] Bentabet N., Boucherit-Otmani Z., Boucherit K., Ghaffour K. (2014). Preliminary phytochemical study of leaves and roots of *Fredolia aretioides*. endemic plant of Algeria. *Der Pharma Chemica*.

[42] Israili Z. H., Lyoussi B. (2009). Ethnopharmacology of the plants of genus *Ajuga*. *Pakistan Journal of Pharmaceutical Sciences*.

[43] Boulfia M., Lamchouri F., Khabbach A., Zalaghi A., Assem N., Toufik H. (2018). An ethnopharmacological evaluation of Moroccan medicinal plants of the middle atlas and pre-rif of the province of Taza. *Journal of Chemical and Pharmaceutical Research*.

[44] Khabbach A., Libiad M., Ennabili A. (2011). Plant resources use in the province of Taza (north of Morocco). *ProEnvironment*.

[45] Casacchia T., Sofo A., Casaburi I., Marrelli M., Conforti F., Statti G. A. (2017). Antioxidant. enzyme-inhibitory and antitumor activity of the wild dietary plant *Muscari comosum* (L.) Mill. *\International Journal of Plant Biology*.

[46] Coelho G. D. P., Martins V. S., Amaral L. V., Novaes R. D., Sarandy M. M., Gonçalves R. V. (2016). Applicability of isolates and fractions of plant extracts in murine models in type II diabetes: a systematic review. *Evidence-Based Complementary and Alternative Medicine*.

[47] Tanenbaum M., Bonnefond A., Froguel P., Abderrahmani A. (2018). Physiopathologie du diabète. *Revue Francophone des Laboratoires*.

[48] Figueiredo-González M., Grosso C., Valentão P., Andrade P. B. (2016). *α*-Glucosidase and *α*-amylase inhibitors from Myrcia spp.: a stronger alternative to acarbose?. *Journal of Pharmaceutical and Biomedical Analysis*.

[49] Daglia M. (2012). Polyphenols as antimicrobial agents. *Current Opinion in Biotechnology*.

[50] Jaberian H., Piri K., Nazari J. (2013). Phytochemical composition and *in vitro* antimicrobial and antioxidant activities of some medicinal plants. *Food Chemistry*.

[51] Jafri L., Saleem S., Ul-Haq I., Ullah N., Mirza B. (2014). *In vitro* assessment of antioxidant potential and determination of polyphenolic compounds of *Hedera nepalensis* K. *Arabian Journal of Chemistry*.

[52] Sroka Z., Cisowski W. (2003). Hydrogen peroxide scavenging. antioxidant and anti-radical activity of some phenolic acids. *Food and Chemical Toxicology*.

